# The Institutional Library

**Published:** 1917-01-06

**Authors:** 


### THE INSTITUTIONAL LIBRARY.

**Arboreal Man.** By F. Wood Jones, M.B., D.Sc. (London : Edward Arnold. 1916. Pp. 224. 8s. 6d. net.)

"It has often been assumed that the anatomist is incapable of making any real contribution towards the knowledge of the origin of Man, since he treats, and rather prides himself on treating, his material as though, to use Huxley's well-known expression, it were sent from some other planet, preserved, it may be, in a cask of rum." This reproach has certainly been removed by the extremely interesting and very able book before us. Dr. Wood Jones is not content, as some anatomists are, merely to describe the anatomical structure of man. He is not content, as other anatomists are, merely to compare the details of the anatomical structure of man with those of the structure of other animals and to point out the homologues. He is not content, as yet other anatomists are, to trace the evolution of an organ in the phylogeny of man, or of the other animal in which the organ is found, and to show the changes through which it has passed from the rudiment to the fully developed organ. He takes a much wider and more comprehensive view. He has made it his task to do these things, not as an end to be pursued for its own sake, but as a means of discovering how and why the changes came about, and especially what environmental conditions have determined them. His aim is to show that in the case of man all that is distinctively human in his structure, and not only in his structure, but in his capacities, abilities, habits, and conduct, are the results, direct and indirect, of the assumption of arboreal life by his very remote ancestors at a very early stage in his phylogeny. Given the arboreal habit assumed at a sufficiently early, plastic, and undifferentiated stage in the phylogeny, and provided the specialisation of structure and function to this habit was not carried too far, all the rest follows, and the assumption of the status of man was not only inevitable, but could not have been attained in any other way. That is Dr. Wood Jones' thesis, and he certainly proves it to the satisfaction of his readers. His account of the gradual evolutionary progress, and of the many blind alleys that lead off from the forthright path, blind alleys into which so many forms that might have been ancestors of man have strayed and reached a stage of evolution very highly specialised, but on that account quite stationary, is fascinating, and has never been so clearly or well displayed.

It is commonly assumed that man is descended from a quadrupedal mammalian form, that he has turned ninety degrees through a vertical circle into the upright position. Dr. Wood Jones shows that if ever this change took place, it took place long before the ancestors of man were mammalian. It was in the amphibian or reptilian stage that he first began to climb, and the habit of climbing, provided it remains true climbing with a grasp and does not become mere clinging by the nails or claws, necessitates an increasingly upright position. At first grasping equally with all four extremities, man's first step in evolution is that the fore limbs become more and more adapted to grasping, the hind limbs more and more adapted to supporting; hence more mobility of the fore limb, more stability of the hind. The greater mobility of the fore limb, and the greater power, delicacy, and applicability of grasp lead directly to the recession of the snout, for the teeth are no longer needed for grasping, and things to be smelt can be carried to the nose instead of being sought after by the nose. The recession of the snout brings the eyes into the same plane, and makes binocular vision, and therefore stereoscopic vision, possible, and so the series of consequential changes proceeds. It is a mistake to suppose that man first assumed the erect position when he began to walk. His climbing ancestors were erect in their arboreal habitat, as his nearest arboreal relatives are now. Long before they could walk erect they hung erect by their hands, swung themselves erect from branch to branch, and sat erect. All those modifications in the suspension of the viscera that adapted them to the erect position were made, therefore, long before man or his anthropoid ancestor began to walk. Dr. Wood Jones shows, further, how the limitation of the family which now causes us such heart-searchings was a necessary consequence of the arboreal habit, for an arboreal mother pregnant with a numerous litter would be deprived of her necessary activity, and after the birth would not have limbs enough to carry them about with her. The book is an essay, not only in evolution, but also in logical deduction. It is well written and well illustrated, and is, as we have shown, of very general as well as of very great interest.

**Home Care of Consumptives.** By Roy L. French, M.A. With twenty-seven Illustrations. Sm. 8vo. Pp. 219+v. (New York and London : G. P. Putnam's Sons. 1916.)

Mr. French has written a sensible little book on his subject. Apparently in America there is a considerable class too poor for the private, and too rich for the public, sanatorium, whose consumptives are doctored at home. In default of example, these are given some excellent precept, not the worst of it the advice of choosing a medical man. "Some of the older men regard tuberculosis as almost always hopeless, and consequently manifest little interest in their cases"; "If your doctor makes an examination through your clothes, consult another physician"—these remarks are true enough. A device which is much more used in America than here is the "sleeping porch," which is an attachment to the exterior of a house over a bedroom window. The price may be as low as £10. As an out-of-door annexe to the ordinary bedroom such an addition would be most helpful in the home treatment of a bedfast case of tuberculosis. One or two points are, of course, exceptionable. On p. 15 the supine posture is recommended in hæmoptysis. This is an error, for three-quarters of all such hæmorrhages occur when the patient is lying down. Sitting up against a bed-rest is much the best. An examination of every organ in the body, with chemical and microscopic examination of the urine and blood-pressure tests, would be remarkably cheap at "about five dollars." And much of the chapter on food preparation could be found in any cookery book. However, most of the book is sound and practical—commonplace adjectives enough, but rarely applicable to the literature of tuberculosis.

**Handbook for Wives and Mothers in India.** By Dr. Mildred Staley. Second Edition. 1916. (Calcutta : Thacker, Spink and Co. Pp. 344. Price 5s. net.)

This book of advice for nurses and mothers in India is very well printed and produced, and on the whole the information it contains is judiciously selected and accurate. There is, too, a good deal less of sentimentality and twaddling than books of this scope usually contain. This may be due to the fact that it is written by a medical woman. The author commits herself in various sections of the book to several statements whose truth is highly dubious, to say the least of it; and we think a more courageous attitude towards the practice of early rising after parturition (a method made in Germany, and universally condemned elsewhere), towards the "twilight sleep" propaganda, and towards several other innovations which have caught the public fancy through copious advertisement, would have been useful to the readers for whom the book is intended. A fuller index is also much to be desired.

**Clinical Methods.** By ROBERT HUTCHINSON, M.D., F.R.C.P., and HARRY RAINY, M.D., F.R.C.P. Ed. Sixth Edition. (Cassell and Co. 1916. Pp. 664. Price 10s. 6d. net.)

THIS is one of the few standard medical text-books which have got such a reputation, and have so thoroughly deserved it, that they have only to be properly revised at intervals to be beyond the reach of rivalry or effective competition. Fortunately, successive editions of this valuable monograph are called for every three or four years, and the periodical revisions have been carried out as carefully and thoroughly as was the original compilation. Perusal of the pages of this sixth edition reveals the same admirable excellence that has been familiar ever since the first issue. The most captious of critics would find little to grumble at; but he *might* possibly complain that the drawing representing the membrana tympani is a poor one; that the description of the operation of lumbar puncture wants rewriting; and that tracings illustrating auricular fibrillation, sinus arrhythmia, and other forms of cardiac irregularity might well replace the not very instructive diagrams on pp. 198-203.

**Crowley's Hygiene of School Life.** By C. W. HUTT, M.A., M.D., D.P.H. (London: Methuen and Co., Ltd. 3s. 6d. net.)

THE earlier edition of Dr. Crowley's text-book had an important influence on the development of the "child welfare" movement in this country, and the present version, extensively revised and brought up to date by Dr. C. W. Hutt, of Brighton, should prove of the highest value to those interested, from any standpoint, in the physical regeneration of the race. The authors write not so much from the point of view of pioneers in the science of hygiene as applied to child life, as for administrators of existing regulations, and within these limits they have done their work with conspicuous success. A comprehensive, but always well-balanced, view of the school medical officer's functions is sketched in the introduction, then item by item we are shown how these duties ought to be, and actually are being, carried out. There are few omissions of any importance, the subject of cleansing being the most surprising. Reference to any of the chapters on special departments of school work, such as school clinics, baths, open-air schools, will place the reader in command of a precise statement of their history, functions, and limitations, illustrated by plans and photographs of the best specimens yet designed, and minute practical suggestions as to their working. The result is a volume indispensable to the school medical officer, school nurse, or member of an education committee, but also deeply interesting to medical practitioners outside the school service. Dr. Hutt has had unusual experience in the handling of abnormal children, and his sections on nervous, backward, epileptic, and mentally defective children contain much fresh material which may prove startling to some who only meet this class of child in the consulting-room and are unaware of what the school can do in the way of amelioration. Like most other authorities on school hygiene, but unlike too many public health administrators, the writers conclude, after an elaborate review of the evidence, that schools play a negligible part in the spread of all infectious diseases, save measles, and even here the effect is not great enough to justify the cry for exclusion of all children under five. A closely reasoned appeal is made for some public educational provision for these babies, whose needs are so commonly misunderstood and cannot possibly be supplied in the working-class home. A helpful feature of the book is the numerous appendices embodying the chief Acts and Regulations under which the school doctor has to work, and including the Binet Simon tests for intelligence.

**Hospital Days.** By "PLATOON COMMANDER." (London: T. Fisher Unwin. 1916. Price 2s. 6d. net.)

WE have not had the pleasure of reading "Platoon Commander's" account of his adventures in the firing-line; but if it is as full of geniality, observation, tolerance, and humour as is "Hospital Days," it must be one of the best books the trenches have given us. In this latter work the author recounts his varied experiences as a casualty, from the moment he was wounded until his convalescence was completed. In a series of racy chapters he describes the whole system of medical aid, from the regimental medical officer and bearers, through the Field Ambulance, the Clearing Station, the Base Hospital, to the various hospital and convalescent establishments in England. Doctors, orderlies, nurses, matrons, and V.A.D.s may all see themselves here as their patients see them; and surely no friendlier critic, no "casualty" more willing to make allowances and to see other people's point of view, could possibly be asked for. The whole story is told with a simplicity, a straightforwardness, an apparent artlessness, that leave a very pleasant impression on the reader, and may cause some people to overlook the real literary touch which the author so deftly gives his pages. This is very much a book for the public at large, as well as for hospital people of both kinds—that is, the patients and the various attendants whose object is to promote their recovery. It deserves a high place amongst current war literature.

**With the Russian Wounded.** By TATIANA ALEXINSKY. (London: T. Fisher Unwin. 2s. 6d.)

IN what capacity the authoress (a political refugee in Paris) worked on a hospital train with the Russian wounded is not quite clear. Her husband states in the preface that she is a doctor, but her patients addressed her as "Sister," and she wore the uniform of a Red Cross nurse. The period was that of the German advance into Poland, and the book contains vivid sketches of that terrible time. The casual way in which the authoress obeyed her chief or not in the matter of treatment or discipline gives a lurid idea of some of the difficulties in the path of the hospital reformer, doctor or matron, but there is something to be said for the capacity of the sympathetic Russian for finding a way round the most hidebound regulations. And so with the women volunteers, of whom Madame Alexinsky met many; with no medical examination to pass, it was simply a question of persuading someone to hand over a uniform and equipment, while Authority looked the other way. A pleasant, readable book, though the letters of gratitude might have been curtailed with advantage.

